# MIC16 gene represents a potential novel genetic marker for population genetic studies of *Toxoplasma gondii*

**DOI:** 10.1186/s12866-016-0726-3

**Published:** 2016-06-08

**Authors:** Wen-Ge Liu, Xiao-Pei Xu, Jia Chen, Qian-Ming Xu, Si-Long Luo, Xing-Quan Zhu

**Affiliations:** State Key Laboratory of Veterinary Etiological Biology, Key Laboratory of Veterinary Parasitology of Gansu Province, Lanzhou Veterinary Research Institute, Chinese Academy of Agricultural Sciences, Lanzhou, Gansu Province 730046 People’s Republic of China; College of Animal Science and Technology, Anhui Agricultural University, Hefei, Anhui Province 230036 People’s Republic of China; Science and Technology College, Shenyang Agricultural University, Fushun, Liaoning Province 113122 People’s Republic of China; Jiangsu Co-innovation Center for the Prevention and Control of Important Animal Infectious Diseases and Zoonoses, Yangzhou University College of Veterinary Medicine, Yangzhou, Jiangsu Province 225009 People’s Republic of China

**Keywords:** *Toxoplasma gondii*, MIC16, Sequence diversity, Genetic marker, PCR-RFLP

## Abstract

**Background:**

The zoonotic agent *Toxoplasma gondii* is distributed world-wide, and can infect a broad range of hosts including humans. Microneme protein 16 of *T. gondii* (TgMIC16) is responsible for binding to aldolase, and is associated with rhomboid cleavage and presence of trafficking signals during invasion. However, little is known of the TgMIC16 sequence diversity among *T. gondii* isolates from different hosts and geographical locations.

**Results:**

In this study, we examined sequence variation in MIC16 gene among *T. gondii* isolates from different hosts and geographical regions. The entire genomic region of the MIC16 gene was amplified and sequenced, and phylogenetic relationship was reconstructed using Bayesian inference (BI) and maximum parsimony (MP) based on the MIC16 gene sequences. The results of sequence alignments showed two lengths of the sequence of MIC16 gene among all the examined 12 *T. gondii* strains: 4391 bp for strains TgCatBr5 and MAS, and 4394 bp for strains RH, TgPLH, GT1, PRU, QHO, PTG, PYS, GJS, CTG and TgToucan. Their A+T content ranged from 50.30 to 50.59 %. A total of 107 variable nucleotide positions (0.1–0.9 %) were identified, including 29 variations in 10 exons and 78 variations in 9 introns. Phylogenetic analysis of MIC16 sequences showed that typical genotypes (Type I, II and III) were able to be grouped into their respective genotypes. Moreover, the three major clonal lineages (Type I, II and III) can be differentiated by PCR-RFLP using restriction enzyme *Pst* I.

**Conclusions:**

Phylogenetic analysis and PCR-RFLP of the MIC16 locus among *T. gondii* isolates from different hosts and geographical regions allowed the differentiation of three major clonal lineages (Type I, II and III) into their respective genotypes, suggesting that MIC16 gene may provide a novel potential genetic marker for population genetic studies of *T. gondii* isolates.

**Electronic supplementary material:**

The online version of this article (doi:10.1186/s12866-016-0726-3) contains supplementary material, which is available to authorized users.

## Background

*Toxoplasma gondii* is an important zoonotic pathogen which can infect all warm-blooded animals and humans. It is estimated that approximately one-third of human population in the world has been infected with *T. gondii* [1–3]. Toxoplasmosis causes several clinical syndromes such as chorioretinitis, encephalitis and systemic infections, particularly seriously in pregnant hosts and immuno-compromised individuals such as those with HIV/AIDS [4–6]. Also, toxoplasmosis can lead to considerable economic losses in livestock industry [7–9].

*T. gondii* isolates from different geographical locations and hosts have been grouped into 15 haplogroups that collectively define six major clades by PCR-restriction fragment length polymorphism (PCR-RFLP) [10, 11]. Despite that genotyping *T. gondii* strains using polymorphisms has been focused on 12 markers including SAG1, SAG3, BTUB, GRA6, c29-2, L358, PK1, 5′-SAG2, 3′-SAG2, alt. SAG2, C22-8 and Apico, additional gene (s) may contribute to some distinct *T. gondii* genotypes [12]. The full knowledge of genetic diversity in *T. gondii* is a key to better understand pathogenicity and epidemiological patterns, and thus to explore a new way for vaccination, treatment and diagnosis of toxoplasmosis.

Microneme proteins (MICs) of *T. gondii* play key roles in *T. gondii* survival and the invasion process, and thus affecting host cell signaling, as well as participating in the binding to host cell receptors [13–15]. Among those MICs, the microneme protein 16 of *T. gondii* (TgMIC16) is responsible for binding to aldolase, is associated with rhomboid cleavage, and thus involved in the trafficking signals during invasion [16]. Although the major functions of TgMIC16 were uncovered, little is known of the sequence variation in MIC16 gene among *T. gondii* isolates.

Therefore, the objectives of the present study were to examine sequence variation in MIC16 gene among 12 *T. gondii* strains from different hosts and geographical locations, as well as to assess whether MIC16 gene can provide a potential marker for population genetic studies of *T. gondii* by phylogenetic analysis and PCR-RFLP.

## Methods

### Genomic DNA samples of *T. gondii*

A total of 12 genomic DNA samples of *T. gondii* were used in this study (Table 1). These *T. gondii* DNA samples had been genotyped in our previous studies [17–19]. The DNA samples of the reference strains GT1, MAS, TgCatBr5, PTG, CTG and TgToucan were kindly provided by Associate Professor Chunlei Su at the Department of Microbiology, the University of Tennessee, Knoxville, USA.

**Table 1:** Details of *Toxoplasma gondii* isolates used in this research

No.	Isolate	Host	Geographical location	Genotype^a^
1	RH	Human	France	Reference, Type I, ToxoDB #10
2	TgPLH	Pig	Henan, China	Type I, ToxoDB #10
3	GT1	Goat	United States	Reference, Type I, ToxoDB#10
4	MAS	Human	France	Reference, ToxoDB#17
5	TgCatBr5	Cat	Brazil	Reference, ToxoDB#19
6	PRU	Human	France	Type II, ToxoDB #1
7	QHO	Sheep	Qinghai, China	Type II, ToxoDB #1
8	PTG	Sheep	United States	Reference, Type II, ToxoDB#1
9	PYS	Pig	Panyu, China	ToxoDB #9
10	GJS	Pig	Jingyuan, Gansu, China	ToxoDB #9
11	CTG	Cat	United States	Reference, Type III, ToxoDB#2
12	TgToucan	Toucan	Costa Rica	Reference, ToxoDB#52

^a^based on the results of Zhou et al. [17, 19] and Su et al. [18]

### PCR amplification

The entire genomic sequence of the MIC16 gene was amplified by PCR using a pair of specific primers (forward primer: 5′-ATGGTTGTTTCCTGTCTCTGTAC-3′; reverse primer: 5′-TTAGAGGTAGTTGTCCCGTGTCC-3′) designed based on the MIC16 gene sequence of *T. gondii* GT1 strain (TGGT1_289630, http://www.toxodb.org/toxo/). The amplification reaction was carried out in a volume of 25 μl containing 10 mM Tris–HCl (pH 8.4), 50 mM KCl, 3 mM MgCl2, 250 μM each of dNTP, 0.2 μM of each primer, 100–200 ng of template DNA, and 0.25 U *La Taq* polymerase (TaKaRa). Amplification of DNA samples from individual isolates was carried out in a thermocycler (Bio-Rad, Hercules, CA, USA) under the following conditions: the initial denaturation at 95 °C for 5 min, followed by 34 cycles consisting of 95 °C for 1 min (denaturation), 64 °C for 45 s (annealing for the two primers), 72 °C for 4 min 30 s (extension for the two primers), and a final extension step was at 72 °C for 10 min. Each (5 μl) amplification was carried out by electrophoresis on a 1 % (w/v) agarose gel with ML5000 DNA ladder marker (TaKaRa), stained with GoldenView™ and photographed using a gel documentation system (UVPGelDoc-It™ Imaging System, Cambridge, UK) [20].

### Sequencing of the MIC16 amplicons

To ensure the accuracy of MIC16 sequences from different *T. gondii* strains, the PCR products of MIC16 gene were purified according to manufacturer’s recommendations (Wizard™ PCR-Preps DNA Purification System, Promega, USA), and then ligated with the pGEM-Teasy (Promega, USA). Thereafter, positive plasmids were transformed into the JM109 competent cells (Promega, USA). Following PCR screening, the positive colonies were sequenced by GenScript (Nanjing) Co., Ltd.

### Sequence analysis and phylogenetic reconstruction

The obtained MIC16 gene sequences from different *T. gondii* strains were aligned using Multiple Sequence Alignment Program, Clustal X 2.11 [21], and sequence variation was determined among the examined *T. gondii* strains. The phylogenetic reconstructions based on the MIC16 sequences from different *T. gondii* strains were performed by Bayesian inference (BI) and maximum parsimony (MP). BI analyses were conducted with four independent Markov chains run for 10,000,000 metropolis coupled MCMC generations, sampling a tree every 10,000 generations in MrBayes 3.1.1 [22]. The first 250 trees were omitted as burn-in and the remaining trees were used to calculate Bayesian posterior probabilities (PP). MP analysis was performed using PAUP* 4.0b10 [23]. Bootstrap support for MP tree was calculated from 1000 bootstrap replicates with 10 random additions per replicate [24].

### PCR-RFLP

PCR-RFLP was used to further assess whether MIC16 gene sequence is a potential marker for genotyping *T. gondii* isolates. The amplified MIC16 fragments were digested with restriction enzyme *Pst* I for 8 h at 37 °C in the appropriate buffer, and then inactivated at 80 °C for 20 min according to the manufacturer’s instructions (NEB). The restriction fragments were analyzed by electrophoresis on 1 % agarose gel stained with GoldenView™ and photographed using a gel documentation system (UVP GelDoc-It™ Imaging System, Cambridge, UK).

## Results

### Sequence analysis

All the positive transformants of the MIC16 gene were approximately 4000 bp in length on 1 % (w/v) agarose gel (not shown). The MIC16 gene sequences were 4391 bp in length for strains TgCatBr5 and MAS; and they were 4394 bp in length for strains RH, TgPLH, GT1, PRU, QHO, PTG, PYS, GJS, CTG and TgToucan. Their A+T content varied from 50.30 to 50.59 %. Alignment of the obtained MIC16 sequences from 12 *T. gondii* strains revealed nucleotide polymorphisms at 107 sequence variation positions (0.1–0.9 %), including 78 variations in 9 introns, 29 variations in 10 exons (Additional file 1: Figure S1). In addition, a total of 20 transversions (A<−>T/C<−>G/T<−>G/A<−>C), 76 transitions (C<−>T /A<−>G), 7 deletions and 4 inserts were identified in MIC16 gene (Additional file 1: Figure S1).

**Fig. 1:**
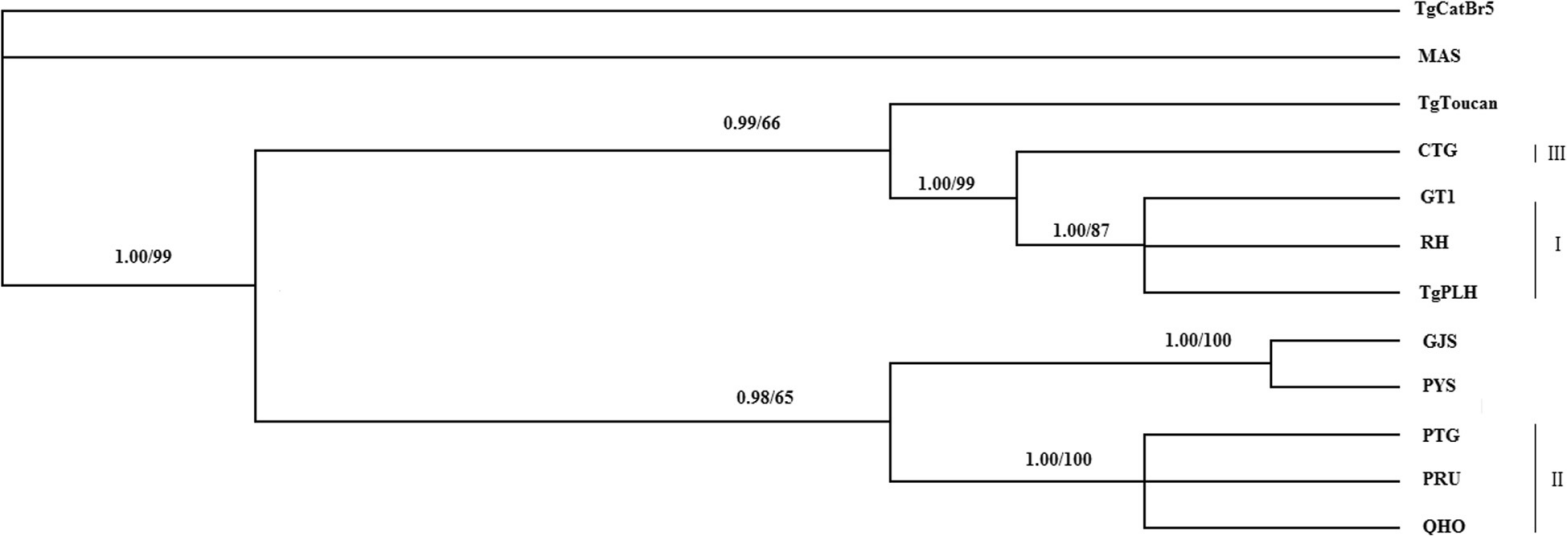
Phylogram of 12 *Toxoplasma gondii* strains determined by analysis of the entire sequences of the MIC16 gene. The tree was built by Bayesian inference (BI) and maximum parsimony (MP) analysis. The numbers along branches show bootstrap values resulting from different analyses: BI/MP

Sequences analysis of polymorphisms in the three references genotypes (RH/GTI, PRU/QHO/PTG, CTG) revealed the existence of polymorphic restriction sites. Using PCR-RFLP method, digestion of the amplification products with *Pst* I allowed the differentiation between genotype I, II, and III (Fig. 1).

### Phylogenetic analysis of *T. gondii* strains based on MIC16 sequences

**Fig. 2:**
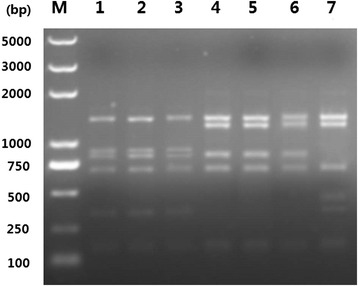
PCR-RFLP analysis of MIC16 genomic sequences of *Toxoplasma gondii* isolates in 1 % agarose gel using restriction endonucleases *Pst* I. Lane M represents DNA size marker 5000. Lanes 1–7 represent *T. gondii* Type I (GTI, RH, TgPLH), Type II (PRU, QHO, PTG), Type III (CTG) strains respectively. Refer to Table 1 for isolate information

By phylogenetic reconstruction based on MIC16 sequence data of all 12 strains, we have obtained the phylogenetic tree (Fig. 2). Phylogenetic analysis revealed three major clusters, which are corresponding to classical genotypes (I, II, III) respectively, and Type III are clustered more closely with type I than with other strains. Atypical strains TgToucan were phylogenetically linked to Type III, and thus the both strains GYS and PYS were phylogenetically linked to Type II.

## Discussion

In the present study, we determined the entire genome region of the MIC16 locus for 12 *T. gondii* strains among different host and geographical origins, and determined genetic diversity between *Toxoplasma* strains based on MIC16 gene sequences. It has been found that there were a total of 107 variable positions distributed in the genome sequence of the MIC16 locus among 14 isolates, including 29 variations in 10 exons and 78 variations in 9 introns. These results suggested higher sequence variations in MIC16 gene than that in MIC4, MIC13 and eIF4A genes [25–27]. The analyses of MIC16 gene in both nucleotides and amino acids among 12 *T. gondii* strains showed high ratio of non-synonymous to synonymous polymorphisms, emphasized that MIC16 was undergoing positive selection together with some other loci, such as GRA5, GRA6 and ROP17 [24, 28, 29].

With the purpose of analyzing the evolutionary relationship among the 12 examined *T. gondii* isolates, the phylogenetic tree was established (Fig. 1) based on the MIC16 sequence using BI and MP methods. Phylogenetic analysis showed that *T. gondii* strains corresponding to classical genotypes (I, II, III) were grouped into their respective clusters separately, which were consistent with the results from other genetic markers including GRA6 and GRA5 genes [24, 28].

Moreover, the three major clonal lineages (Type I, II and III) can be differentiated by digestion of MIC16 PCR products using endonuclease *Pst* I (Fig. 2), which further suggested that the MIC16 locus may be a potential new genetic marker for PCR-RFLP genotyping of *T. gondii* strains. However, some atypical strains including TgCatBr5, TgToucan, PYS, GJS and MAS can not be distinguished from the three major lineage types by PCR-RFLP (not shown), suggesting that PCR-RFLP genotyping with a single locus is not enough for the identification of non-clonal genotypes.

## Conclusions

The present study examined sequence variation in MIC16 and demonstrated the existence of sequence variability in MIC16 gene among the examined *T. gondii* strains from different hosts and geographical localities. Phylogenetic analysis and PCR-RFLP genotyping of the examined *T. gondii* strains suggest that MIC16 gene may provide a potential marker for population genetic studies of *T. gondii* isolates, and further research sampling more isolates from different hosts and geographical separations need to be done to verify this possibility.

## Abbreviations

AIDS, acquired immune deficiency syndrome; BI, Bayesian inference; HIV, human immunodeficiency virus; MP, maximum parsimony; PCR, polymerase chain reaction; PCR-RFLP, PCR-restriction fragment length polymorphism; TgMIC16, *Toxoplasma gondii* microneme protein 16

## Additional file

Additional file 1: Figure S1. Alignment of MIC16 gene sequences of 12 *Toxoplasma gondii* strains. Lower case letter “a” indicates the base changes in exons; Lower case letter “b” indicates the base changes in introns. (JPEG 87 kb)
