# Supplementary figures and images for: MIC16 gene represents a potential novel genetic marker for population genetic studies of Toxoplasma gondii
Source: BMC Microbiol. 2016 Jun 8;16:101. doi: 10.1186/s12866-016-0726-3 (PMC4898453; doi:10.1186/s12866-016-0726-3)

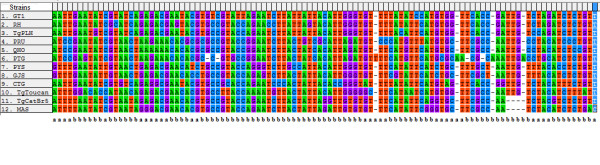

Supplement: Additional file 1: Figure S1. — Alignment of MIC16 gene sequences of 12 Toxoplasma gondii strains. Lower case letter “a” indicates the base changes in exons; Lower case letter “b” indicates the base changes in introns. (JPEG 87 kb) [file 12866_2016_726_MOESM1_ESM.jpeg]
